# Lehren der COVID-19-Pandemie: Erfahrungen von Intensivpflegefachpersonen während der COVID-19-Pandemie: eine qualitative explorative Studie

**DOI:** 10.1007/s00063-024-01120-4

**Published:** 2024-03-08

**Authors:** Elena Camenzind, Luzia Vetter, Matthias Thomas Exl, Marie-Madlen Jeitziner

**Affiliations:** 1 Universitätsklinik für Anästhesiologie und Schmerzmedizin, Universitätsspital Bern (Inselspital), Bern, Schweiz; 2 Pflegeentwicklung und -qualität, Luzerner Kantonsspital, Luzern, Schweiz; 3 Universitätsklinik für Intensivmedizin, Universitätsspital Bern (Inselspital), Bern, Schweiz; 4 Departement Public Health (DPH), Pflegewissenschaft – Nursing Science (INS), Universität Basel, Basel, Schweiz

**Keywords:** Erfahrung, Intensivpflegefachpersonen, Skill-Grade-Mix, Arbeitsbelastung, Qualitative Forschung, Experience, Critical care nurses, Skill-grade mix, Workload, Qualitative research

## Abstract

**Hintergrund:**

Wenn die Belastung für Intensivpflegefachpersonen zu hoch wird, kann dies Folgen auf die persönliche Gesundheit und auf die Versorgung der Patient*innen haben. Während der Coronavirus Krankheit 2019(Coronavirus disease 2019 [COVID-19])-Pandemie waren Intensivpflegefachpersonen mit neuen und dynamischen Veränderungen konfrontiert.

**Ziel der Arbeit:**

Ziel war es, Erfahrungen der Intensivpflegefachpersonen bez. der getroffenen Ad-hoc-Maßnahmen und der empfundenen physischen und psychischen Belastung während der COVID-19-Pandemie zu beschreiben.

**Methode:**

Es wurde eine Querschnittserhebung mittels Onlinebefragung in 2 Krankenhäusern durchgeführt. Die offen formulierten Fragen zu den Herausforderungen während der COVID-19-Pandemie wurden inhaltsanalytisch nach Mayring analysiert.

**Ergebnisse:**

Insgesamt haben 179 Intensivpflegefachpersonen teilgenommen. Die 4 gebildeten Kategorien umfassen: „den eigenen Ansprüchen an die Pflegequalität nicht gerecht werden“, „Unsicherheiten im beruflichen sowie privaten Alltag“, „erhöhte Verantwortung bei fehlender Entlastung“ und „insuffiziente Bewältigungsstrategien für physische und psychische Belastungen“.

**Diskussion:**

Intensivpflegefachpersonen benötigen Strukturen und Prozesse, die sie in Situationen hoher Arbeitsbelastung unterstützen. Der Schwerpunkt sollte dabei auf den Umgang mit den eigenen Ansprüchen an die Pflegequalität sowie den Einsatz von potenziell entlastenden Maßnahmen liegen.

**Zusatzmaterial online:**

Zusätzliche Informationen sind in der Online-Version dieses Artikels (10.1007/s00063-024-01120-4) enthalten.

## Abstract (en)

**Background:**

When the workload for critical care nurses becomes too high, this can have consequences for both personal health as well as patient care. During the COVID-19 pandemic, critical care nurses were confronted with new and dynamic changes.

**Objective:**

The aim of this study was to describe the experiences of critical care nurses regarding the ad hoc measures taken and the perceived physical and psychological burden experienced during the coronavirus disease 2019 (COVID-19) pandemic.

**Methods:**

This was a cross-sectional study conducted at two hospitals using an online survey. The open questions addressing the challenges faced during the COVID-19 pandemic were subjected to content analysis according to Mayring.

**Results:**

A total of 179 critical care nurses participated in the online survey. From the results, the following four categories were developed: “not meeting one’s own quality of care requirements,” “uncertainties in everyday professional and private life,” “increased responsibility with lack of relief,” and “insufficient coping strategies for physical and psychological burden.”

**Conclusion:**

Critical care nurses require structures and processes which support them in situations of high workload. The focus should be on the self-imposed requirements of quality of care as well as potentially relieving measures.

Physische und psychische Belastungen gehören für Intensivpflegefachpersonen zum Alltag [29]. In Krisensituationen wie der COVID-19-Pandemie können diese Belastungen sprunghaft ansteigen. Maßnahmen zur Entlastung werden ad hoc entwickelt und etabliert. Eine Evaluation der Erfahrungen mit diesen Belastungen und Maßnahmen findet jedoch selten statt. In diesem Beitrag beschreiben wir, wie Intensivpflegefachpersonen während der COVID-19-Pandemie diese Belastungen und Maßnahmen erlebt haben.

## Hintergrund

Wenn die Belastung für Intensivpflegefachpersonen zu hoch wird, kann dies elementare Folgen auf die persönliche Gesundheit und auf die Versorgung der Patient*innen haben [13, 15]. Die Auswirkung der Belastung auf die Intensivpflegefachpersonen hat die Coronavirus Krankheit 2019(Coronavirus disease 2019 [COVID-19])-Pandemie eindrücklich gezeigt [16]. Angebote zur Entlastung und Unterstützung der Intensivpflegefachpersonen wie beispielsweise Personalverschiebung oder Einsatz von Hilfskräften, Care Teams oder unentgeltliche Verpflegung wurden ad hoc entwickelt und etabliert.

Ein weiteres Angebot war beispielsweise der Einsatz von Pflegefachpersonen der Normalstationen auf den Intensivstationen (ICU), denen das Arbeitsgebiet wenig bekannt war oder die fachliche Qualifikation zur Verantwortungsübernahme fehlte. Dadurch formierten sich während der COVID-19-Pandemie kurzfristig neuartige Formen der interprofessionellen Teamarbeit zwischen Pflegefachpersonen untereinander [6]. Gerade interprofessionelle Teamarbeit und das Kennen der Mitarbeitenden sind aber ein Erfolgsschlüssel der Intensivmedizin [10, 24]. Diese Zusammenarbeit stellte Pflegefachpersonen, pflegerisches und ärztliches Führungspersonal, aber auch die Institutionen vor unterschiedliche Herausforderungen. Diese sind: kompetent bei schweren und lebensbedrohlichen Krankheitsverläufen in einem ungewohnten Team handeln, veränderte Rolle und Funktion der erfahrenen Intensivpflegefachpersonen, wie Führungsfunktionen über Pflegefachpersonen der Normalstationen, in 12-Stunden-Schichten oft personalmäßig unterbesetzt arbeiten [4, 16], Zusatzbelastung durch einen hohen unvorhersehbaren Arbeitsaufwand und eine enorme Verantwortung [5, 8]. Dazu waren Pflegefachpersonen konfrontiert mit großem Leid und eigenen Ängsten, Sorgen [3] und einer geringfügigen Kriseninterventionskompetenz in den Gesundheitssystemen. Fachliche Überforderung, physische, psychische und emotionale Belastung wurden als Folgen beschrieben [12, 16–18].

Ziel dieser Studie war es daher, Erfahrungen der Intensivpflegefachpersonen bez. der getroffenen Ad-hoc-Maßnahmen und der empfundenen physischen und psychischen Belastung während der COVID-19-Pandemie zu beschreiben.

## Methode

Es wurde eine Querschnittserhebung mittels einer Onlinebefragung diplomierter Expert*innen in Intensivpflege mit einem Nachdiplomstudiengang sowie Studierender dieser Fachrichtung durchgeführt (aufgrund der besseren Lesbarkeit schreiben wir folgend Intensivpflegefachpersonen). Die Erhebung fand von Mitte bis Ende Mai 2021, kurz nach Abflachung der zweiten Welle, in der Schweiz statt. Die Onlinebefragung wurde in 2 Schweizer Spitälern durchgeführt (Spital A = Universitätsspital, Spital B = Kantonsspital). Bis zum Zeitpunkt der Befragung herrschte eine veränderte Arbeitssituation aufgrund der COVID-19-Pandemie. Es wurden 12,5 h-Schichten eingeführt sowie ein Ferienstopp verhängt. Auch unterstützende Angebote wie das Care Team und unentgeltliche Verpflegung für das Personal wurden eingeführt. Das Care Team besteht aus Fachpersonen unterschiedlicher Konfessionen und steht Patient*innen und Angehörigen sowie dem Gesundheitsfachpersonal rund um die Uhr zur Verfügung. Ihre Aufgabe ist die seelisch-geistige und religiös-spirituelle Unterstützung in Krisensituationen. Zudem wurden Pflegefachpersonen mit und ohne Nachdiplomstudium in Anästhesiepflege sowie ehemalige Intensivpflegefachpersonen kurzfristig rekrutiert und eingesetzt. Zur Befragung wurde das Onlinebefragungstool (enuvo GmbH, Pfäffikon, Schweiz) eingesetzt. Dieses erfüllt alle Anforderungen und Vorschriften der General Data Protection Regulation (GDPR) der Europäischen Union. Nebst validierten Fragebögen zur Resilienz [20] und beruflichen Lebensqualität [28] kamen 11 offene Fragen zum Einsatz. Die Fragen umfassten Herausforderungen im Umgang mit der COVID-19-Pandemie im Berufsalltag sowie im privaten Umfeld. Die Fragen wurden aufgrund von Literatur und Expertise formuliert sowie in einem Pilot bez. Verständlichkeit getestet und befinden sich im Anhang (Anhang 1). Diese Publikation zeigt die Resultate der offenen Fragen, die inhaltsanalytisch nach Mayring (2015; [21]) mit der Analysesoftware f4analyse V3.4.1 (dr. dresing & pehl GmbH, Marburg, Deutschland [2]) ausgewertet wurden. Die Kategorien wurden unabhängig von 2 der beteiligten Forschenden induktiv gebildet. Um das Gütekriterium Intersubjektivität einzuhalten [14], haben die Forschenden die Kategorien überprüft und diskutiert. Die Kategorien wurden bis zum Konsens mehrfach neu zugeteilt und angepasst, bis das endgültige Kategoriensystem entwickelt wurde. Die Archivierung einer transparenten und nachvollziehbaren Dokumentation des Prozesses hat laufend stattgefunden.

### Ethik

Die Kantonale Ethikkommission Bern, Schweiz erteilte eine Nichtzuständigkeit (Req-2021-00846). Die Teilnahme an der Befragung war freiwillig, anonym und konnte jederzeit abgebrochen werden. Die Intensivpflegefachpersonen wurden schriftlich informiert und konnten persönlich weitere Informationen bei den Forschenden einholen.

## Ergebnisse

Insgesamt haben 179 Intensivpflegefachpersonen auf die offenen Fragen geantwortet. Die Studienteilnehmenden haben eine Frage bis alle Fragen beantwortet (Median = 8 „range“ = 1–11). Der Umfang der Antworten reichte von einzelnen Stichworten bis zu ausführlichen Situationsbeschreibungen. Die Tab. 1 gibt einen Überblick zu den demografischen und beruflichen Charakteristika der Studienteilnehmenden.

**Tab. 1:** Demografische Daten

Demografische Daten	Total	Spital A	Spital B
Geschlecht (w/m/d)	*n* = 179 (159/19/1)	*n* = 128 (115/12/1)	*n* = 51 (44/7)
Alter (Median ± Standardabweichung (SD))	35 ± 9,802	35,5 ± 9,638	34 ± 10,153
„Range“ (Jahre)	21–63	25–62	21–63

*w* weiblich, *m* männlich, *d* divers, *n* Anzahl, *SD* Standardabweichung

Aus den Befragungen konnten 4 Kategorien gebildet werden. Die Kategorien umfassen: den eigenen Ansprüchen an die Pflegequalität nicht gerecht werden, Unsicherheiten im beruflichen sowie privaten Alltag, erhöhte Verantwortung bei fehlender Entlastung sowie insuffiziente Bewältigungsstrategien für physische und psychische Belastungen.

### Den eigenen Ansprüchen an die Pflegequalität nicht gerecht werden

Eine zentrale Kategorie ist „den eigenen Ansprüchen an die Pflegequalität nicht gerecht werden“. Eine Definition der Pflegequalität und was die Einschränkung bedeutet, wurde von niemandem benannt. Es wurde stets von der eigenen Wahrnehmung der schlechter gewordenen Qualität und den eigenen Ansprüchen an diese berichtet.

Die Intensivpflegefachpersonen konnten nicht mehr ihren persönlichen oder professionellen Werten und Ansprüchen genügen. Dadurch war ihr gewohnter Standard, der als Pflegequalität erlebt wird, nicht mehr möglich und sie empfanden ein Nichtgerechtwerden an ihre Ansprüche. So fühlten sich die Intensivpflegefachpersonen physisch, psychisch, emotionell und moralisch in ihrem Berufsverständnis eingeschränkt, da sie nicht mehr die Pflegequalität anbieten konnten, die sie für notwendig hielten.

„… Ich denke, die Versorgungsqualität hat sicherlich gelitten. Aber die größte Herausforderung finde ich, dass man nach der Schicht so oft mit einem schlechten Gefühl Feierabend machte, weil man seinen Ansprüchen oder den Qualitätsstandards nicht gerecht werden konnte.“ (Frage 2, Absatz 97)

„Die Frustration niemandem gerecht zu werden und oft am wenigsten den Patienten hat mich sehr beschäftig und zeitweise stark frustriert. Ich konnte die Betreuung und Pflege nicht so umsetzen, wie ich es mir von mir gewohnt bin, und meine Pflegequalität litt, was ich schrecklich fand ….“ (Frage 1a, Absatz 4)

„Sichere Pflege war öfters nicht möglich und ich musste Maßnahmen/Tätigkeiten weglassen, die man nicht weglassen dürfte. Dies führte zu einer psychische Dauerbelastung und Angst, es könnte etwas passieren und Konsequenzen haben.“ (Frage 1a, Absatz 84)

### Unsicherheiten im beruflichen sowie privaten Alltag

Die Kategorie „Unsicherheiten im beruflichen sowie privaten Alltag“ bezieht sich auf die Veränderungen des Arbeitsalltags während der COVID-19-Pandemie. Die unbekannte Betreuungsintensität und der ungewisse Krankheitsverlauf sowie das fehlende Intensivpflegefachpersonal waren eine große Herausforderung.

„… Der Verlauf einer COVID-19-Erkrankung und die damit verbundenen Konsequenzen, wie lange Aufenthaltsdauer und viele Rückschritte oder Stagnation bei der Genesung der Patienten, waren und sind mit Abstand das Herausforderndste an der ganzen Pandemie für mich.“ (Frage 1a, Absatz 10)

Für die Intensivpflegefachpersonen war aufgrund der Besuchsrestriktionen auch die veränderte Angehörigenbetreuung belastend. Durch die eingeschränkten Besuchszeiten ergaben sich Konflikte und Unstimmigkeiten zwischen den Angehörigen und den Intensivpflegefachpersonen sowie unter den Intensivpflegefachpersonen. Die Besuchsrestriktionen wurden beispielsweise nicht von allen Intensivpflegefachpersonen einheitlich umgesetzt. Aufgrund der eingeschränkten Besuchsmöglichkeiten, die durch Isolation oder Quarantäne der Angehörigen verstärkt wurden, gab es weniger direkten Angehörigenkontakt. Die persönliche Beziehung zwischen Angehörigen und Intensivpflegefachpersonen konnte teilweise kaum aufgebaut und erhalten werden.

„… Angehörigenbetreuung! Brachte mich an den Rand des Wahnsinns. Die ständigen Diskussionen und sich rechtfertigen zu müssen, als böse hingestellt zu werden. Dann wurden die Besuchsregelungen nicht von allen Pflegenden gleich strikt umgesetzt. So versuchten die Angehörigen oft, uns zu manipulieren und wir wurden angelogen.“ (Frage 1d, Absatz 87)

Auch das Risiko für eine Übertragung und Erkrankung an SARS-CoV-2 löste viele Ängste und Unsicherheiten in der Bevölkerung und unter Gesundheitsfachpersonen aus. Die Intensivpflegefachpersonen hatten Angst davor, die Erkrankung in ihr privates Umfeld zu tragen. Dies wurde dadurch verstärkt, dass zu Beginn der COVID-19-Pandemie unklar war, ob ausreichend Schutzmaterial vorhanden ist und ob sich die Mitarbeitenden entsprechend gegen eine Ansteckung schützen können.

„… Ich fand es herausfordernd, dass es im privaten und beruflichen Umfeld viele Unklarheiten bezüglich der Pandemie gab. Am meisten fürchtete mich der Gedanke, dass ich und mein persönliches Umfeld an COVID erkrankt.“ (Frage 1d, Absatz 64)

### Erhöhte Verantwortung bei fehlender Entlastung

Eine weitere Kategorie war die „erhöhte Verantwortung bei fehlender Entlastung“. Aus der höheren Arbeitsbelastung und der veränderten Teamzusammensetzung ergab sich ein größerer Verantwortungsbereich. Die Intensivpflegefachpersonen übernahmen die Verantwortung über den Pflegeprozess von mehr Patient*innen als gewohnt. Zwar wurden die Patient*innen durch Hilfskräfte betreut, die Verantwortung für den Pflegeprozess lag aber weiterhin bei den Intensivpflegefachpersonen. Die meisten Hilfskräfte, die die Patient*innen betreuten, konnten die Verantwortung nicht tragen und somit war der Druck auf die Intensivpflegefachpersonen erhöht. Dadurch entlasteten die Hilfskräfte das Intensivpflegefachpersonal nur bedingt.

„Die zweite Welle habe ich deutlich anstrengender erlebt. Viele Ausfälle und dadurch magere Teamkonstellationen. Viel Verantwortung, teilweise hatte ich die Verantwortung über 4 Patient*innen zusammen mit Studierenden und klinikexternen Mitarbeitenden. … Wir als Intensivpflegende hatten kaum Zeit, dies abzufangen.“ (Frage 1a, Absatz 107)

Diese Diskrepanz wurde verstärkt, durch die fehlenden bekannten Teamzusammensetzungen in den Teams der Intensivpflegefachpersonen. Die Hilfskräfte waren meistens nicht bekannt und auch deren Kompetenzen oft unklar.

„… Die teilweise täglich wechselnden Teamkonstellationen, oftmals kannte man die wenigsten Mitarbeitenden, es brauchte immer wieder Neuorientierung.“ (Frage 1d, Absatz 54)

Weiter war die Studierendenbetreuung kaum möglich und die Intensivpflegefachpersonen fühlten sich dafür verantwortlich. Die praktische Ausbildung der Studierenden konnte kaum stattfinden und die komplexen Betreuungssituationen führten zur persönlichen und fachlichen Überforderung der Studierenden. Diese Situation unterhielt die erhöhte Arbeitsbelastung und den größeren Verantwortungsbereich der Intensivpflegefachpersonen.

„… Ich fand vor allem die Betreuung der Studierenden zunehmend schwierig, was sich auf ihren Praxis-Theorie-Transfer negativ auswirkt. Sie mussten oftmals einfach funktionieren ohne genügend Support zu erhalten.“ (Frage 2, Absatz 110)

### Insuffiziente Bewältigungsstrategien für physische und psychische Belastungen

Die letzte Kategorie sind die „insuffizienten Bewältigungsstrategien für physische und psychische Belastungen“. Physische und psychische Belastungen gehören für Intensivpflegefachpersonen zum Alltag, doch in der COVID-19-Pandemie kamen die individuellen Bewältigungsstrategien an ihre Grenzen. Die Intensivpflegefachpersonen berichteten über psychische Symptome wie Konzentrationsprobleme, Mangel an Motivation oder Frust. Auch physische Symptome, wie Erschöpfung und große Müdigkeit, sind zu dieser Zeit aufgetreten. Diese Symptome wurden im Zusammenhang mit der veränderten Arbeitssituation durch die COVID-19-Pandemie beschrieben und der hohen Arbeitsbelastung ohne eine Entlastung des Arbeitsaufwands.

„… Das Personal lief sowieso schon am Rande des Unmöglichen und diese Wechsel waren für alle (auch Patienten) eigentlich nicht zumutbar. Der Frust und das Gefühl, nicht verstanden zu werden, stieg sehr.“ (Frage 1a, Absatz 113)

„… Ich bin erschöpfter und genervter, habe keine Geduld mehr, das kenn ich von mir nicht. Die Pandemie und auch die Arbeitsbelastung (keine Flauten gehabt), die stets hoch ist, macht sich spürbar.“ (Frage 4, Absatz 85)

Die individuelle Belastung im privaten Umfeld stieg ebenfalls an, weil die COVID-19-Pandemie zu einer veränderten sozialen und privaten Situation führte. So wurden Freizeitangebote massiv eingeschränkt und sonstige Möglichkeiten zum Ausgleich waren nicht mehr vorhanden.

„… Mir fehlten viele Möglichkeiten für den persönlichen Ausgleich, da Sportzentren etc. geschlossen waren.“ (Frage 1d, Absatz 24)

## Diskussion

Unsere Studie zeigt, dass Intensivpflegefachpersonen durch die COVID-19-Pandemie einer sehr großen Arbeitsbelastung ausgesetzt waren und eine erhöhte Verantwortung übernehmen mussten; die initialen Unklarheiten bezogen auf die SARS-CoV-2-Erkrankung, die persönliche Gesundheit, die unbekannten therapeutischen Möglichkeiten und die ständig wechselnden Teamzusammensetzungen als bedeutsame Herausforderungen.

Insbesondere dass die Intensivpflegefachpersonen ihren eigenen Ansprüchen an die Pflegequalität nicht gerecht wurden, wirkte für sie belastend. Verschiedene Studien zu moralisch belastenden Situationen konnten negative Auswirkungen auf die Pflegequalität, Patient*innensicherheit und die Fachpersonen aufzeigen; dies sobald nicht entsprechend den professionellen Werten und Maßstäben betreut werden kann [9, 25]. Intensivpflegefachpersonen in unserer Studie sahen sich gezwungen, gegen ihre professionellen und persönlichen Werte und Standards zu handeln. Sie konnten aus ihrer Sicht nicht mehr das Richtige tun, mussten priorisieren oder auch rationieren. Smith und Godfrey (2002; [27]) vermuten, dass ein Zusammenhang zwischen dem Sein einer guten Pflegefachperson und dem Tun des Richtigen besteht. Die Verpflichtung und das Einhalten der Tugenden betrifft damit nicht nur die Handlungen, sondern auch den Charakter der Pflegefachperson [27]. Die Intensivpflegefachpersonen empfanden die von ihnen geleistete Pflegequalität nicht mehr angemessen und konnten dies nicht mit dem Berufsverständnis und der Integrität einer guten Pflegefachperson vereinbaren. Die Standards und Therapiemöglichkeiten konnten nicht wie gewohnt durchgeführt werden. Der Berufsalltag der Intensivpflegefachpersonen ohne die COVID-19-Pandemie war nicht mehr so, wie er normalerweise ist [22]. Weiter ist in den untersuchten Institutionen nicht klar, wie Standards in außergewöhnlichen Situationen ad hoc angepasst werden und wie viel zusätzliche Verantwortung Pflegefachpersonen übernehmen müssen.

Während der COVID-19-Pandemie herrschten Unsicherheiten im beruflichen sowie privaten Alltag. Zwischenmenschliche Interaktionen waren im interprofessionellen Team aber auch mit Patient*innen sowie deren Angehörigen verändert oder gar minimiert. Schilling et al. (2022; [26]) zeigen in ihrem systematischen Review die Wichtigkeit von Interaktionen während der COVID-19-Pandemie auf. Dabei gilt es, die unterschiedlichen Fähigkeiten der Mitarbeitenden zu betonen und zu stärken, Rollen und Verantwortlichkeiten zu klären, den formellen kontinuierlichen Informationsaustausch zu erleichtern sowie die informellen Kommunikationsmöglichkeiten zu entwickeln. In unseren Ergebnissen wird beschrieben, wie zu Beginn der COVID-19-Pandemie Unklarheiten im Umgang mit der SARS-CoV-2-Erkrankung Ängste und Unsicherheiten unter Intensivpflegefachpersonen ausgelöst haben. Dies wurde durch die Frage, ob ausreichend Schutzmaterial vorhanden sei, verstärkt. Diese Ängste und Unsicherheiten waren auch nach der zweiten Welle noch vorhanden. Nikbakht Nasrabadi et al. (2022; [23]) zeigen in ihrem systematischen Review und Metasynthese die Wichtigkeit eines adäquaten Supports durch Schulung sowie einem Mangel an Pflegepersonal und Ausrüstung proaktiv zu begegnen.

In unserer Studie berichteten die Intensivpflegefachpersonen über die Problematik mit erhöhter Verantwortung bei fehlender Entlastung. Diese ergab sich durch die höhere Arbeitsbelastung und die zusätzliche Verantwortung für die durch Hilfskräfte betreuten Patient*innen. Die interprofessionelle Teamarbeit bildet ein Schlüsselelement der modernen Intensivmedizin und sorgt für positive Patient*innenoutcomes [10, 11]. In unserer Studie zeigte sich, dass gerade die interprofessionelle Teamarbeit durch Hilfskräfte, Unklarheiten über Kompetenzen oder ständig wechselnde Teamzusammensetzungen eine große Herausforderung darstellte. Eine Lösung für diese Belastung hätte eine klare Kompetenz- und Verantwortungszuteilung sein können. Zusätzlich wären beständige Teams ebenfalls förderlich gewesen; die Intensivpflegefachpersonen hätten sich dann auf bekannte Ressourcen stützen können, ohne wieder eine „fremde“ Person mit ihren Fähigkeiten einschätzen zu müssen. Die Problematik mit erhöhter Verantwortung bei fehlender Entlastung kam auch in der Ausbildung der Studierenden zum Vorschein. Anderson, Moxham und Broadbent (2018; [1]) haben in ihrer Studie herausgefunden, dass Pflegefachpersonen motiviert sind, Studierende zu unterstützen, weil sie glauben, es sei das Richtige. Dies kann zu Zeiten einer hohen Arbeitsbelastung und Personalmangel nicht mehr erfüllt werden. Unsere Studienergebnisse zeigen weiter, dass die Intensivpflegefachpersonen mit der von ihnen geleisteten Studierendenbetreuung unzufrieden waren.

Internationale Studien zeigen, dass die COVID-19-Pandemie mit beachtlichen physischen, psychischen, emotionalen und moralischen Folgen für die Intensivpflegefachpersonen einherging [7, 16, 19]. In unserer Studie deckt sich dies mit der Kategorie der insuffizienten Bewältigungsstrategien für physische und psychische Belastungen. Die 12,5 h-Schichten und keine Ferien forderten das Intensivpflegefachpersonal physisch und psychisch. Auch unterstützende Angebote, wie das Care Team oder unentgeltliche Verpflegung, konnten dies auf Dauer nicht lindern. Gewohnte Strukturen, Supportdienste, Arbeitsabläufe, Betreuungsschlüssel sowie Arbeitsplanung mussten kurzfristig angepasst oder konnten nicht mehr angewendet werden. Möglicherweise verschärfte die Pandemie bestehende Probleme und spiegelte eindrücklich künftige Herausforderungen für Fachpersonen und Gesellschaft wie z. B. Fachpersonenmangel, Betreuungsschlüssel, Arbeitsbelastung, Berufsverständnis und Care-Ethik. Unsere Studie zeigt, dass neben der Quantität die Qualität (Qualifikation) der Pflegefachpersonen eine wesentliche Anforderung für eine zielgerichtete Patient*innenversorgung darstellt.

## Limitationen

Unsere Studie birgt verschiedene Stärken und Schwächen. Die Studie wurde in 2 unterschiedlichen Spitälern durchgeführt, in denen die Intensivpflegefachpersonen unterschiedliche Erfahrungen während der COVID-19-Pandemie gemacht haben. Die Befragung erfolgte zeitnah, wodurch die erlebten Erfahrungen bei der Befragung noch präsent und nachvollziehbar waren. Die Befragung erfolgte online, dadurch konnten zeitnah, benutzerfreundlich und einfach Daten erhoben werden, jedoch bestand das Risiko von Mehrfachantworten und die erschwerte Kontrollierbarkeit der Antworten. Als Limitation kann betrachtet werden, dass vor allem Intensivpflegefachpersonen mit einer vertieften Ausbildung und Qualifikation in Intensivpflege befragt wurden. Offen bleibt, welche Erfahrungen Pflegefachpersonen auf den ICU ohne spezifische Ausbildung und Qualifikation für dieses Setting gemacht haben.

## Fazit für die Praxis

Intensivpflegefachpersonen benötigen Strukturen und Prozesse, die es ihnen ermöglichen, mit einem veränderten Berufsverständnis und einer den eigenen Ansprüchen nicht gerecht werdenden Pflegequalität umzugehen. Strukturen und Prozesse können beispielsweise durch die klare Kommunikation von Kompetenzen und Verantwortung geschaffen werden. Fallbesprechungen bieten eine Unterstützung zur Auseinandersetzung mit dem Berufsverständnis und ermöglichen eine differenzierte Reflexion. Der zeitnahe Einsatz von Ethiker*innen, Psycholog*innen oder von Peers zusammen mit dem interprofessionellen Team könnte hierfür zielführend sein. Weiter gilt es, die interprofessionelle Teamarbeit und den Einsatz von Hilfskräften zu diskutieren und zu fördern sowie Konflikte zu thematisieren. Die physischen sowie psychischen Symptomen von Intensivpflegefachpersonen sollten erfasst und professionell begleitet werden. Hierfür ist noch weitere Forschung notwendig.

## Supplementary Information

Fragebogen Mitarbeitenden Befragung COVID-Pandemie
