# Supplementary material for: Lessons from the COVID-19-Pandemic: Experiences of critical care nurses during the COVID-19 pandemic: a qualitative explorative study
Source: Med Klin Intensivmed Notfmed. 2024 Mar 8;120(1):65–70. [Article in German] doi: 10.1007/s00063-024-01120-4 (PMC11761982; doi:10.1007/s00063-024-01120-4)
Supplement: Supplementary file 1 — Fragebogen Mitarbeitenden Befragung COVID-Pandemie [file 63_2024_1120_MOESM1_ESM.docx]

**Fragebogen Mitarbeitenden Befragung COVID-Pandemie**

In der Online-Umfrage kam, neben validierten Fragebögen zur Resilienz und beruflichen Lebensqualität, 11 offene Fragen zum Einsatz.

1. Wie hast Du die 1. bzw. 2. COVID-Welle erlebt? Bitte positive und negative Aspekte nennen, z. B. 12h Schichten, etc.
2. Wie hast Du den Umgang mit Über- bzw. Minus- Zeit in der 1. bzw. 2. COVID Welle erlebt?
3. Welche Aspekte hast Du während der 1. und der 2. COVID-Welle geschätzt, z. B. bezahlte Mittagspause, freie Getränke und Früchte, etc.?
4. Welche Aspekte waren für Dich während der COVID-Pandemie besonders herausfordernd und was hättest Du benötigt, um diese Herausforderung besser meistern zu können?
5. Wie hast Du die Unterstützung durch das Personal der Intensivstation, bzw. durch das Supportpersonal erlebt?
6. Wie hast Du die interprofessionelle, bzw. interdisziplinäre Zusammenarbeit erlebt?
7. Welche Erwartungen hattest Du an das Supportpersonal bzw. an das Personal der Intensivstation?
8. Wie hat sich die Pflege- und Versorgungsqualität während der COVID-19-Pandemie verändert (1. und 2. Welle) und in welchen Situationen hast Du diese Veränderung besonders festgestellt?
9. Wie hast Du die Rekrutierung für den Einsatz auf der Intensivstation erlebt (1. und 2. Welle)?
10. Wie hast Du die Einführung auf der Intensivstation erlebt?
11. Etwas Anderes, dass ich noch anmerken möchte:
